# Book Reviews

**Published:** 1799-10

**Authors:** 


### CRITICAL RETROSPECT

OF

### MEDICAL AND PHYSICAL LITERATURE.

[FOREIGN AND DOMESTIC.]

*A Treatife on Febrile Difeafes; including intermitting, remitting, and continued Fevers; eruptive Fevers, Inflammations, Hæmorrhagies, and the Profluvia: in which an Attempt is made to prefent at one View, whatever, in the prefent State of Medicine, it is requifite for the Phyfician to know refpecting the Symptoms, Caufes, and Cure of thofe Difeafes. By* A. P. WILSON, M. D. F. R. S. *Ed. Phyfician to the County Hofpital, at Winchefter, &c.* Vol. I. 8vo. pp. 729, *London.* Cadell and Davies.

THE author, in his Preface, gives the following account of his views in undertaking this work. "When I firft turned my attention particularly "to febrile difeafes, I had no view of undertaking fo laborious a work as "that in which I am now engaged. For feveral years, I devoted the whole "of my time to the ftudy of thefe complaints, in order to qualify myfelf "for reading lectures on them, which I did in the fummer of 1796, at "Edinburgh.

"My reafons for making choice of febrile difeafes for the fubject of my "lectures were, that they form the moft important branch of medicine, "and that which is leaft generally underftood. The practice in moft "other difeafes is fimple; but in febrile complaints the fymptoms are "infinitely varied; minute circumftances often point out effential dif-"ferences in their nature, and confequently in the plan of treatment.

"A very infirm ftate of health has obliged me to abandon the plan of "continuing to give lectures, and I am inclined to think that I may render "the refult of my ftudies ufeful to others, in another form.

"With regard to the extent of the work, as far as I can judge, five "volumes will comprehend the whole of my plan. In the fecond, and "part of the third volume, I fhall finifh the firft part, that which treats of "idiopathic fevers; and the fecond part, which treats of the fymptomatic, "will form the remainder of the third and the two laft volumes. The pre-"fent volume, however, forming a treatife on *intermittent, remittent,* and "*continued fevers,* may be regarded as not effentially connected with the "volumes which are to follow."

This firft volume commences with an *Introduction,* which contains the author's *Nofology* of febrile difeafes, not materially differing from that of Dr. CULLEN, but more correct in feveral of the definitions.

The *firft book* is devoted to the confideration of *intermitting* and *remitting fevers.* In the treatment or cure of thefe, he lays down the conduct proper to be purfued during the paroxyfm, and during the apyrexia, or remiffion.

During the cold ftage, he recommends *external* warmth and bliflers, but not internal ftimulants of any kind, unlefs emetics may be comprehended under that title.

In the hot ftage, the indication is to produce a copious perfpiration as foon as poffible; which is effected by removing irritation, as that of bile in the ftomach; ftomach ; by diluents ; by fudorifics ; by fupporting the action of the heart and arteries ; and laftly, by moderating excitement. The means of fulfilling thefe are then explained, with an account of the modus operandi of the remedies.

During the apyrexia, the author gives directions relative to the diet and exercife, at confiderable length ; and then examines the feveral medicines and the beft mode of adminiftering them, viz. the barks, aromatics, Fowler's folution of arfenic, opium, mercury, &c.

The *fecond book* treats of *continued fevers*, in which Dr. Wilfon confiders the fymptoms, caufes, crifes, prognofis, contagion, &c. and under the head of *Proximate Caufe*, he examines Dr. CULLEN's hypothefis. This hypothefis, the invention of HOFFMAN, was flightly varied by Dr. Cullen, and has been fo generally received by his pupils, that we apprehend our author will difpleafe fome of thefe, by the flight notice he takes of it, and the little importance he affigns to it.

The Brunonian doctrine is explained at greater length ; its defects and errors are pointed out ; its merit candidly appreciated and commented upon ; and feveral means of correcting and extending its application fuggefted : we believe, however, that many of BROWN's partizans will not agree with our author in feveral of his objections to that fyftem, nor in fome of his propofed improvements.

Under the head of treatment or *cure* of continued fever, Dr. Wilfon *firft* confiders the means of ftopping a fever at its commencement, by inducing a *crifis* : and *fecondly*, the treatment when we fail to induce a *crifis* at its commencement. On both thefe points, Dr. Wilfon has exhibited the practice of the beft authors ; and his regular reference to them, through his whole work, confiderably enhances its value to the young ftudent ; to whom we warmly recommend it as the beft fyftematic introduction and guide we have feen.

---

*Lectures on Diet and Regimen : being a fyftematic inquiry into the moft rational means of preferving Health and prolonging Life ; together with Phyfiological and Chemical Explanations, calculated chiefly for the ufe of families, in order to banifh the prevailing abufes and prejudices in Medicine. The fecond Edition, improved and enlarged, with confiderable additions.* By A. F. M. WILLICH, M. D. 8vo. 708 pages. (Price Nine Shillings in boards, or half a Guinea on fine paper) *London.* Longman and Rees. 1799.

The tendency of this work is fufficiently obvious from its title ; and as it will not be expected that *we* fhall here enter upon a critical examination of its merits or demerits, we truft to difcharge our duty to the unprejudiced reader, by giving a fhort analyfis of its various contents, together with a few extracts.

In an " Advertifement" prefixed to the firft edition of thefe " *Lectures*," the author informs the public, that, with the exception of the eighth chapter " Of Evacuations," and the ninth " Of the Sexual Intercourfe," they were delivered in the months of January and February, 1798, at Bath, and in the fubfequent fpring at Briftol, to numerous and refpectable audiences. In the compofition of this comprehenfive work, he acknowledges his obligations to many Englifh and German writers, and adds the following exordium : " Should the rules and cautions interfperfed throughout, tend in the fmalleft degree to increafe the knowledge of the inquifitive, diffuade the unwary from injurious habits, or refcue the fenfualift from the brink of deftruction, the exertions of the author will be amply compenfated."

An

An "Analytical Table of Contents" is prefixed to the work, and an Alphabetical Index is added, to facilitate occasional reference.

In a general "Introduction," from page 25 to 98, the author explains the design of this publication; takes a cursory view of the general laws of Nature; and investigates the origin and causes of disease. The doctrine of temperaments he illustrates with some practical remarks of Professor SOEMMERING; from these we extract the following passages:

"There is," says that learned Professor, "a certain line observable in all the more perfect animals, by which Nature is regulated in performing the functions of body and mind; in preserving or impairing the health, and in exerting all those energies of life, on which the happiness of the creature depends. This line is various in different individuals, and the variety cannot be completely explained on the principle of the ancients, by a difference in the qualities of the blood alone; though a human body of a moderate size contain not less than thirty pounds weight of that fluid. Other terms must therefore be substituted for their sanguine, choleric, phlegmatic and melancholy temperaments," p. 40—After having taken a more extensive view of the economy of man, and investigated the remedies suited to the causes productive of a particular disposition of mind and body, Professor Soemmering attempts to classify and fix the characteristic marks of the different temperaments.

"All the modifications of temperaments," says he, "appear to be varieties of the *sanguine* and *phlegmatic*.

1. The sanguine is variable. It is marked by a lively complexion; the vessels are full of blood; and persons of this habit are seldom able to bear great warmth; they are predisposed to inflammations, and possess a high degree of irritability and sensibility. All is voluptuous in this temperament. They are fickle in every thing they undertake; are affable, and soon become acquainted, but as soon forget their friends, and are suspicious of every body. Whatever requires industry they abhor, and hence they make little progress in science, till they advance in age.

"2. The sanguineo-choleric enjoys all the health and serenity of the sanguine, with all the perseverance of the choleric.

"3. In the choleric, the body is soft and flexible, without being dry and meagre, as in the melancholic; the skin has a tint of yellow; the hair is red, the eyes dark and moderately large, with a penetrating expression, and frequently a degree of wildness; the pulse full and quick; the muscular contractions in walking, speaking, &c. are rapid; the bile is copious and acrid, and hence the vermicular motion is active, and the body not liable to costiveness. Persons of this class are particularly fond of animal food. They possess great magnanimity, are fitted for laborious undertakings, and seem born to command.

"He whose temperament is hypochondriacal, is a burthen to himself and others. Persons of this class are subject to diseases of the liver, and hence have a sallow complexion. They are never content with their situation, and are a prey to envy and suspicion.

"The melancholic temperament is marked by a gloomy countenance, small, hollow blinking eyes; black hair; a rigid or tough skin, dry and meagre fibres. The pulse is weak and languid, the bile black, the vermicular motion slow. The perceptions of persons of this disposition are quick, they are fond of contemplation, and are slow in the execution of labour, which they patiently undertake; they bear with resolution the troubles of life; and, though not easily provoked, are nevertheless vindictive.

"The

"The bœotic, or rustic temperament, has many of the qualities of the sanguine, in common with many of those of the phlegmatic. The body is brawny, the muscles have but little irritability, the nerves are dull, the manners rude, and the powers of apprehension weak.

"The gentle temperament is a combination of the sanguine, choleric and phlegmatic. Universal benevolence is the distinguishing character of this class: their manners are soft and unruffled; they hate talkativeness; and if they apply to science, their progress is great, as they are persevering and contemplative. Lastly,

"The phlegmatic class is marked by a soft white skin, prominent eyes, a weak pulse, and languid gait. They speak slowly, are little hurt by the injuries of the weather, and seem born to obey. From their little irritability, they are not easily provoked, and soon return to their natural state of indifference and apathy." p. 47,

(To be continued in our next Number.)

---

*Observations on the diseased and contracted Urinary Bladder, and frequent painful Micturition; with some Cautions respecting the Use of the caustic Bougie, in the Treatment of Strictures in the Urethra; to which are added, Observations on the Schirro-contracted Rectum, &c. By* JOHN SHERWEN, M. D. Member of the Corporation of Surgeons, (1s. 6d.) *London*, Johnson. 1799.

The author justly observes, that the diseased and contracted urinary bladder, in some of its features, nearly resembles the scirrhous rectum, and like that produces a frequent and often fruitless stimulus to expulsion; and though, like the schirrous rectum, it does not admit of being cured, it will sometimes admit of palliation.

After having given an accurate diagnosis of this disease, and investigated the predisposing causes, Dr. Sherwen points out the pathognomonic symptoms, by which it may be distinguished from *calculus*. He strongly recommends the use of the caustic or armed bougie, and faithfully describes the method of applying it with advantage.—His observations on the schirro-contracted rectum are pertinent and original: he illustrates them with the history of a fatal case, from which he draws the practical inference, that clysters, cathartics, and diluents are hurtful in this disease, while relief might be obtained from mechanical means—catheters and bougies. These, according to his directions, ought to be made of horn, or whalebone, smoothly polished; as this substance, by immersion in boiling water, becomes soft and pliant, and will retain its softness some time after it is removed from the boiling water. It will adapt itself to the natural curvature of the pelvis, and should be carried on to the obstructed part slowly, gently, and steadily, with the utmost tenderness and circumspection, but at the same time with sufficient force and resolution.

The "Postscript" contains some useful hints, which we here communicate to our readers:

"Since the publication of the above paper," says Dr. SHERWEN, "I have been consulted in several melancholy cases of this disease, and have, in some instances, promoted a discharge of thin fæces, by the introduction of a rectum probe, made of polished whalebone.

"In one unhappy case, that of the late Mr. HOARE, of Enfield, the purging had existed, and been managed with tolerable comfort, upwards of twenty years; but the gut became at last so much closed and diseased, that that the fæces made a paſſage into the bladder; and, during the laſt month of his life, not a drop was diſcharged except through the penis, from which it was almoſt conſtantly oozing, mixed with urine.

"There are ſymptoms connected with a diſeaſed bladder and rectum, which have been often erroneouſly aſcribed, by medical men of high reputation and real abilities, to an enlargement of the proſtrate gland. It is therefore the duty of every practitioner, ſince that gland lies within the reach of his finger, to take the earlieſt opportunity of examining and aſcertaining its condition. In thoſe caſes, which have fallen under my obſervation, I have moſt frequently found it in a ſtate of extenuation."

NEW MEDICAL PUBLICATIONS IN GERMANY.

